# Regulation of *Brn3b* by DLX1 and DLX2 is required for retinal ganglion cell differentiation in the vertebrate retina

**DOI:** 10.1242/dev.142042

**Published:** 2017-05-01

**Authors:** Qi Zhang, Jamie Zagozewski, Shaohong Cheng, Rajiv Dixit, Shunzhen Zhang, Jimmy de Melo, Xiuqian Mu, William H. Klein, Nadean L. Brown, Jeffrey T. Wigle, Carol Schuurmans, David D. Eisenstat

**Affiliations:** 1 Department of Human Anatomy and Cell Science, University of Manitoba, Winnipeg, Canada R3E 0J9; 2 Department of Medical Genetics, University of Alberta, Edmonton, Canada T6G 2H7; 3 Department of Pediatrics and Child Health, University of Manitoba, Winnipeg, Canada R3A 1S1; 4 Hotchkiss Brain Institute, University of Calgary, Canada T2N 4N1; 5 Department of Biochemistry and Medical Genetics, University of Manitoba, Winnipeg, Canada R3E 3J7; 6 Department of Biochemistry and Molecular Biology, University of Texas M.D. Anderson Cancer Center, Houston, TX 77030, USA; 7 Department of Cell Biology and Human Anatomy, University of California, Davis, CA 95616, Canada; 8 Department of Ophthalmology, University of Manitoba, Winnipeg, Canada R3T 2N2

**Keywords:** Homeobox, Chromatin immunoprecipitation, *In utero* electroporation, *Atoh7*, *Math5*, Mouse

## Abstract

Regulated retinal ganglion cell (RGC) differentiation and axonal guidance is required for a functional visual system. Homeodomain and basic helix-loop-helix transcription factors are required for retinogenesis, as well as patterning, differentiation and maintenance of specific retinal cell types. We hypothesized that *Dlx1*, *Dlx2* and *Brn3b* homeobox genes function in parallel intrinsic pathways to determine RGC fate and therefore generated *Dlx1*/*Dlx2*/*Brn3b* triple-knockout mice. A more severe retinal phenotype was found in the *Dlx1*/*Dlx2*/*Brn3b*-null retinas than was predicted by combining features of the *Brn3b* single- and *Dlx1*/*Dlx2* double-knockout retinas, including near total RGC loss with a marked increase in amacrine cells in the ganglion cell layer. Furthermore, we discovered that DLX1 and DLX2 function as direct transcriptional activators of *Brn3b* expression. Knockdown of *Dlx2* expression in primary embryonic retinal cultures and *Dlx2* gain of function *in utero* strongly support that DLX2 is both necessary and sufficient for *Brn3b* expression *in vivo*. We suggest that ATOH7 specifies RGC-committed progenitors and that *Dlx1* and *Dlx2* function both downstream of ATOH7 and in parallel, but cooperative, pathways that involve regulation of *Brn3b* expression to determine RGC fate.

## Abstract (precis)

**Summary:** *Dlx1*/*2* homeobox genes regulate retinal ganglion cell (RGC) differentiation by directly activating *Brn3b* expression; accordingly, *Dlx1*/*Dlx2*/*Brn3b* triple-knockout mice exhibit near complete RGC loss.

## INTRODUCTION

Six classes of neurons and Müller glial cells are generated from a common retinal progenitor cell (RPC) population, following a precise temporal sequence (Livesey and Cepko, 2001). The first differentiated cells in the mouse are retinal ganglion cells (RGCs), followed by horizontal, cone and amacrine cells. Development of rod photoreceptors, bipolar cells and Müller glia extends into the second postnatal week (Cepko et al., 1996).

Transcription factors (TFs) act intrinsically during different stages of retinogenesis. Homeodomain (HD) and basic helix-loop-helix (bHLH) TFs initiate retinal development and maintain RPC (Ohsawa and Kageyama, 2008). Overexpression of seven eye field TFs in *Xenopus* embryos can induce eye-like structures with functional properties (Viczian et al., 2009).

*Atoh7* (also known as *Math5*) is a bHLH TF essential for RPCs to become RGCs (Brown et al., 2001; Wang et al., 2001; Yang et al., 2003). *Brn3b* (*Pou4f2*) genetically downstream of *Atoh7* is required for the terminal differentiation and survival of most RGCs, but not for their initial specification (Erkman et al., 1996, 2000; Gan et al., 1996; Xiang, 1998). *Brn3b* and *Brn3a* (*Pou4f1*) have overlapping yet distinct roles controlling RGC development and function (Badea et al., 2009). *Isl1*, a LIM-HD TF, under the regulation of ATOH7, defines a distinct but overlapping subpopulation of RGCs with *Brn3b* (Mu et al., 2008; Pan et al., 2008). This *Atoh7*-*Brn3b*/*Isl1* pathway determines a population of RGCs, whereas other RGCs rely on the Distal-less homeobox genes *Dlx1* and *Dlx2* for their differentiation and survival (de Melo et al., 2005, 2008).

Retinas from *Dlx1*/*Dlx2*^−/−^ mice have reduced RGCs due to enhanced apoptosis of late-born RGCs (de Melo et al., 2005). DLX1/DLX2-mediated regulation of the *Dlx5/6* intergenic enhancer and brain-derived neurotrophic factor-mediated TrkB signaling may contribute to the differentiation and survival of RGCs, respectively (de Melo et al., 2008; Zhou et al., 2004).

DLX2 and BRN3B are expressed in distinct but partly overlapping regions in the retinal neuroepithelium (de Melo et al., 2003) (Fig. S1). Furthermore, DLX2 and to a lesser extent DLX1, are expressed in cycling as well as postmitotic RPC (Eisenstat et al., 1999). We hypothesized that *Dlx1* and/or *Dlx2* and *Brn3b* function in parallel intrinsic pathways to determine RGC fate and generated *Dlx1*/*Dlx2*/*Brn3b* triple knockout (TKO) mice. We found almost complete RGC loss with a marked increase in amacrine cells in the ganglion cell layer (GCL). DLX1 and DLX2 were also identified as transcriptional activators of *Brn3b* expression supported by *in utero* retinal electroporation of *Dlx2* and siRNA-mediated knockdown of *Dlx2* in primary embryonic retinal cultures. Taken together, DLX1 and DLX2 are necessary and sufficient for *Brn3b* expression during retinal development.

## RESULTS

### Loss of *Dlx1*/*Dlx2* and *Brn3b* gene function leads to defective RGC specification

**Fig. 1.**
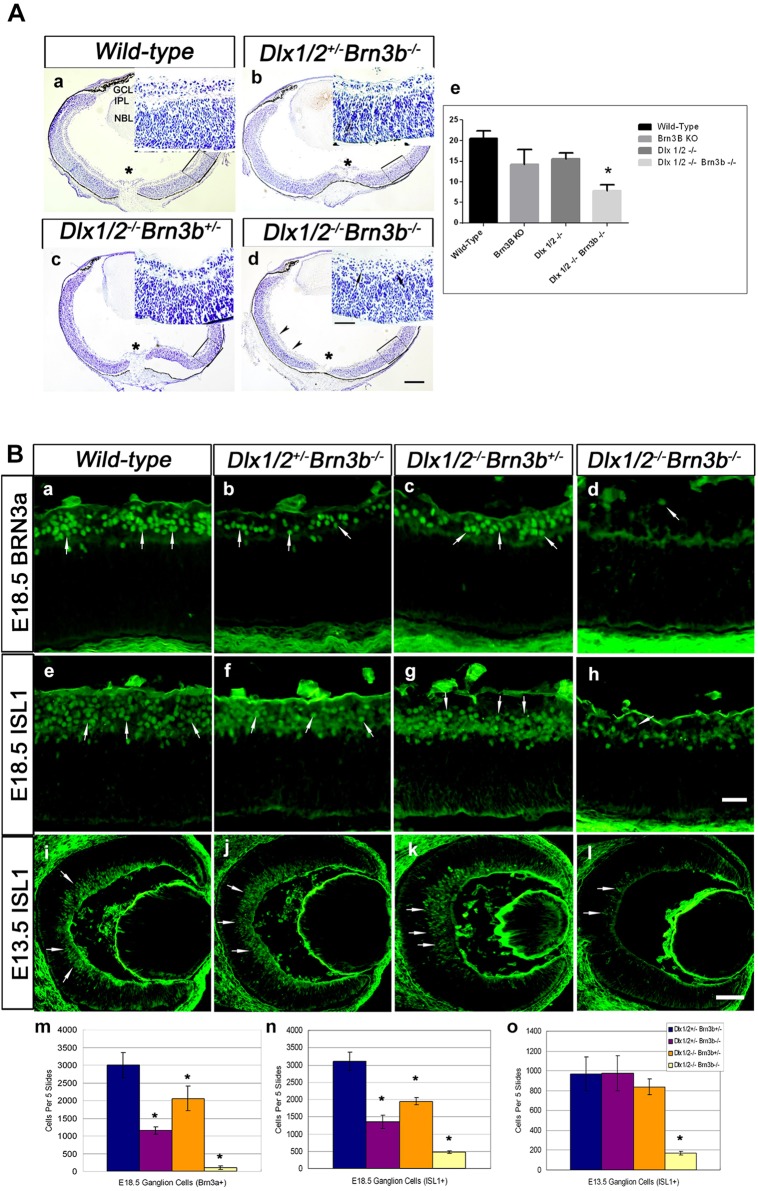
**Severe RGC loss in *Dlx1*/*Dlx2*^−/−^*Brn3b*^−/−^ retinas.** (A) There is a reduced GCL in mutant retinas. Compared with wild type (a), the GCL of the *Dlx1*/*Dlx2*^+/−^*Brn3b*^−/−^ single (b), *Dlx1*/*Dlx2*^−/−^*Brn3b*^+/−^ double (c) and *Dlx1*/*Dlx2*^−/−^*Brn3b*^−/−^ triple (d) KO retinas appear reduced, but no significant difference can be distinguished between the mutants. Near fusion of the GCL and NBL is evident in the TKO (inset in d, arrows) with significant reduction of the IPL (e). Insets show boxed regions at a higher magnification. Asterisk marks the optic nerve head. (Ba-h) Immunostaining with RGC markers BRN3A and ISL1 shows decreased RGCs in all mutant retinas at E18.5; the effect is more severe in the TKO (d; h, arrow). (i-l) Significant loss of ISL1+ RGC is observed only in TKO retinas at E13.5. (m,n) At E18.5, a more severe RGC loss is detected in the TKO (95% reduced BRN3A+ and 84% reduced ISL1+ cells). (o) At E13.5, 81% ISL1+ RGC loss occurs only in TKO retinas. Histograms represent the mean±s.d., *n*=4, *P<0.01. L, lens; NBL, neuroblastic layer; R, retina. Scale bars: 50 μm in Ad, 25 μm in insets; 20 μm in Bh; 50 μm in Bl.

In the *Dlx1/Dlx2* DKO there is 33% loss of late-born RGCs at E18.5, whereas *Brn3b* deletion results in a 60-70% reduction of RGCs in the postnatal retina, depending upon the genetic background. However, neither the *Dlx1/Dlx2* DKO nor the *Brn3b* single knockout (SKO) have defects in other retinal cell classes (de Melo et al., 2005; Erkman et al., 1996; Gan et al., 1996). We hypothesized that the TKO retina would have severe abnormalities in RGC differentiation and survival, with a significantly reduced GCL. *Dlx1/Dlx2/Brn3b* TKO mice die shortly after birth at P0. Unexpectedly, the TKO retina showed only a modestly decreased GCL (Fig. 1Aa,d), whereas the inner plexiform layer (IPL) separating the GCL and NBL was significantly reduced (*P*<0.05, Fig. 1Ad,e).

Few RGCs were detected in the TKO (Fig. 1Bd,h) with ∼95% reduction of BRN3A+ RGCs in the TKO (78.5±14) compared with wild type (2701±148) (*P*<0.01, *n*=4) and ∼80% loss of ISL1+ cells in the TKO (*P*<0.01, *n*=4) (Fig. 1Bm,n). Decreased BRN3A+ cells (37%) and ISL1+ cells (40%) were also observed in *Dlx1*/*Dlx2* DKO retinas (Fig. 1Bc,g,m,n). However, RGC loss in *Brn3b*-null retinas (64% of BRN3A+ cells, 56% of ISL1+ cells) at E18.5 (Fig. 1Bb,f,m,n) did not reach the 70% loss previously reported (Erkman et al., 1996; Gan et al., 1999, 1996). At E16.5, both BRN3A+ and ISL1+ RGCs were diminished in *Brn3b* SKO, *Dlx1*/*Dlx2* DKO and TKO retinas. At E13.5, ISL1 was used to detect RGCs due to low BRN3A expression at this developmental time-point, with 82% reduction of ISL1+ expression, but only in the TKO (*P*<0.01, *n*=4) (Fig. 1Bi-l,o). Similar reductions of ISL+ RGCs were observed in E12.5 TKO retinas (Fig. S2). *Dlx1*/*Dlx2* and *Brn3b* may have redundant functions during early retinogenesis, as neither knockout mouse demonstrated defective early retinal differentiation.

### Increased amacrine cells in the *Dlx1*/*Dlx2*/*Brn3b*-null ganglion cell layer

**Fig. 2.**
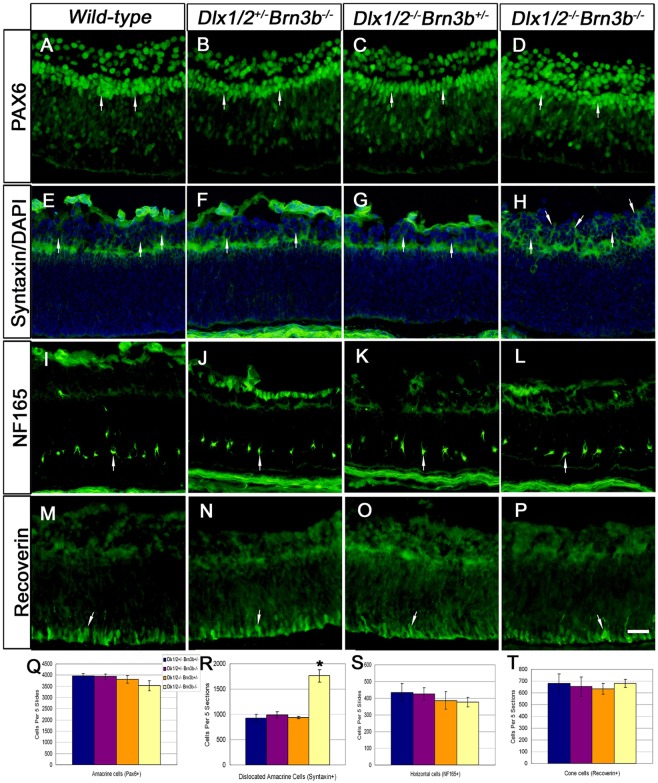
**Increased number of amacrine cells are located in the *Dlx1*/*Dlx2*^−/−^*Brn3b*^−/−^ ganglion cell layer.** (A-D,Q) No significant differences in PAX6+ amacrine cells are identified in the inner NBL in the different mutants (arrows, Q). However, more PAX6+ cells are evident in the TKO GCL (D). (E-H,R) Syntaxin immunostaining reveals increased number of displaced amacrine cells in the TKO GCL (H, arrows), with a 1.8-fold increase in syntaxin+ amacrine cells (R). (I-L,S) The horizontal cell marker NF165 is expressed at expected levels (S) and in the expected position in the TKO (arrow). (M-P,T) Recoverin expression is not significantly different between the mutants and wild type. Data are mean±s.d., *n*=4, *P<0.01. Scale bar: 20 μm.

Amacrine, horizontal and cone cells all have overlapping birthdates with RGCs. PAX6+ cells located in the inner NBL were counted as amacrine cells. No significant difference in PAX6 expression was observed when comparing wild-type with the KO models (Fig. 2A-D,Q). PAX6 is expressed in both RGCs and in displaced amacrine cells in the GCL at E18.5 (Belecky-Adams et al., 1997; de Melo et al., 2003; Inoue et al., 2002). PAX6+ cells in the GCL of *Brn3b* SKO and *Dlx1/Dlx2* DKO were reduced in number (Fig. 2B,C) due to RGC loss (de Melo et al., 2005; Gan et al., 1996). However, in the TKO GCL, there was only minimal reduction in the number of PAX6+ cells (Fig. 2D), supporting the observation of more displaced amacrine cells in the TKO GCL. Syntaxin is present in all amacrine cells but not in RGCs (Barnstable et al., 1985). The number of TKO GCL cells was only partially reduced (Fig. 2H). A significant 1.8-fold increase of syntaxin+ cells was observed in the TKO GCL (1761±122) compared with wild type (930±72) (*P*<0.01, *n*=4) (Fig. 2E-H,R). However, syntaxin+ cells in *Brn3b* SKO and *Dlx1/Dlx2* DKO GCL were not significantly altered.

No significant abnormalities in horizontal cell number or position were observed in TKO retinas (Fig. 2I-L,S). Most cone photoreceptors are born prenatally in mice, but many markers are not detected until P5 (Chow et al., 2001). Recoverin is expressed by cones and cone bipolar cells from E17.5 (Milam et al., 1993). No abnormalities in recoverin+ cell position (Fig. 2M-P) or number (Fig. 2T) were identified.

**Fig. 3.**
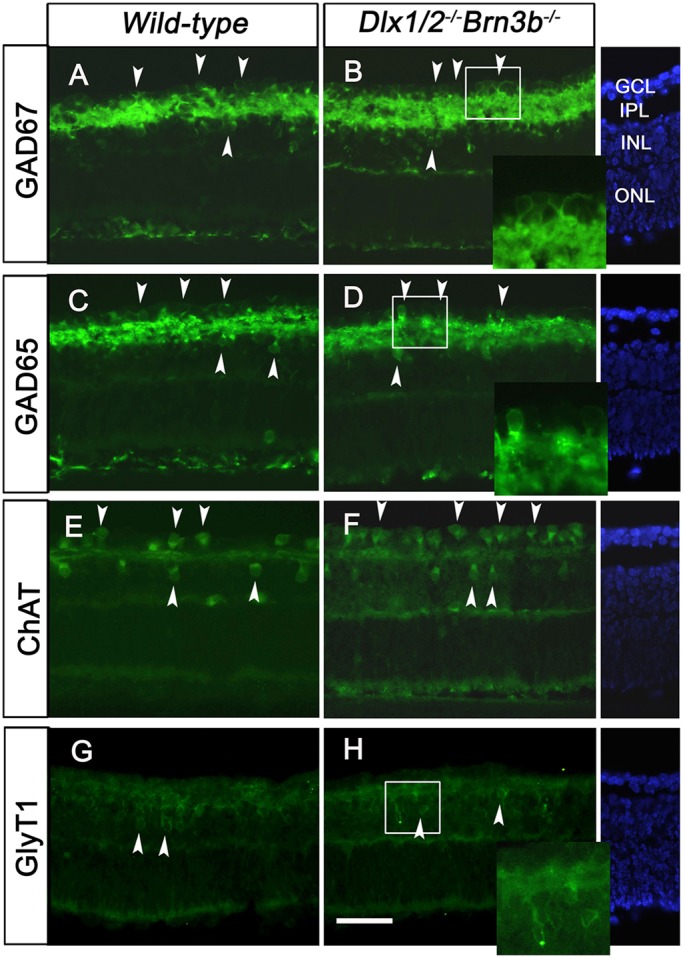
**More cholinergic amacrine cells are located in the GCL of TKO retinal explants.** (A-D) At 7 DIV, GAD65 and GAD67 are highly expressed in the IPL, as well as in the GCL and inner INL of both wild-type and TKO retinas (A-D, arrowheads). (E,F) More ChAT-expressing cells are located in the TKO GCL than in wild-type retina (E,F, arrowheads). (G,H) GlyT1 expression is restricted to the INL of both wild-type and TKO retinas (G,H, arrowheads). Scale bar: 20 μm. Blue staining is DAPI.

To determine the identity of displaced amacrine cells of TKO retinas, we cultured E18.5 TKO and wild-type retina explants for 7 days *in vitro* (DIV7). GABAergic and glycinergic cells represent almost 90% of amacrine cells (MacNeil and Masland, 1998). Glutamic acid decarboxylase (GAD) isoforms, GAD65 and GAD67, were similarly expressed in the IPL and GCL of wild-type and TKO DIV7 retinas (Fig. 3A,B). Starburst cholinergic amacrine cells (expressing choline acetyltransferase, ChAT) are early born GABAergic amacrine cells (Voinescu et al., 2009). Compared with wild-type littermates (Fig. 3E), more ChAT+ cells are observed in the TKO GCL (98.4±9.7 vs 59.5±3.4, *P*<0.01, *n*=4) (Fig. 3F). However, there was no difference in the expression of the glycinergic amacrine cell marker glycine transporter (GlyT1) (Fig. 3G,H). Similarly, there was no difference in expression of the bHLH NEUROD1 across the four genotypes (Fig. S3). Upregulation of ChAT but not GlyT1 in TKO retinas is consistent with increased early born amacrine cells.

**Fig. 4.**
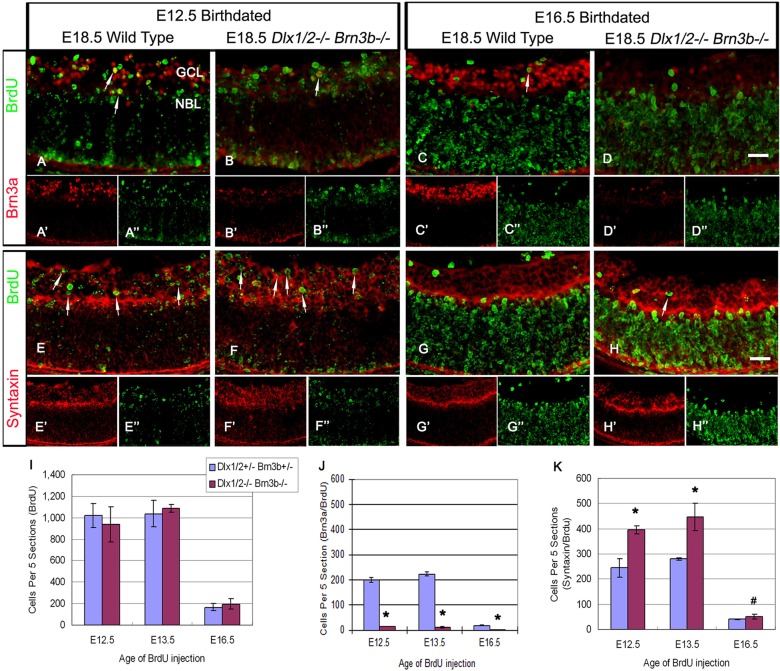
**More retinal progenitors adopt an amacrine cell fate in the *Dlx1*/*Dlx2*^−/−^*Brn3b*^−/−^ mutant GCL.** (A,B,E,F) BrdU pulse labeling at E12.5 and co-expression with BRN3A or syntaxin reveal few RGCs in the TKO (A,B, arrows); more cells differentiate as amacrine cells and migrate to the GCL (E,F, arrows). Most E12.5 BrdU pulse-labeled cells are located in the GCL (B″,F″). (C,D,G,H) BrdU pulses at E16.5 identify more displaced amacrine cells in the TKO, although most BrdU+ cells remain in the NBL. Arrows in C and H mark BrdU+ cells that neither express BRN3A (C) nor syntaxin (H) in the GCL. (I-K) E12.5, E13.5 and E16.5 birth-dated cells identified similar quantities of BrdU+ cells in the GCL of wild-type and TKO retinas (I). At all stages tested, RPCs of the TKO adopt an amacrine rather than a RGC fate in the GCL (J,K). Data are mean±s.d., *n*=4, *P<0.005, ^#^*P*<0.05. Scale bar: 20 μm.

Birth-dating experiments labeled RPC with a single BrdU pulse in pregnant animals at E12.5, E13.5 or E16.5. Most E12.5 and E13.5 birth-dated cells were located in the GCL, without differences in quantity or spatial expression between TKO and wild-type controls (Fig. 4A,B,E,F; data not shown). Few E16.5 birth-dated cells migrated to the GCL; most remain in the NBL (Fig. 4C,D,G,H). BrdU+ cell numbers in the GCL were similar in wild-type and TKO in the E12.5, E13.5 and E16.5 birth-dated retinas (Fig. 4I). Early migration of RPC to the GCL was unaffected in the TKO. Few cells were co-labeled by BRN3A and BrdU in E12.5 birth-dated TKO retinas (14±1 of mutants versus 199±10 of wild type, *P*<0.005, *n*=4; Fig. 4A,B,J). However, more cells were co-labeled with syntaxin and BrdU in the GCL (Fig. 4E,F,K). Similar results were observed in E13.5, as well as in E16.5, birth-dated mutants (Fig. 4C,D,G,H,J,K; data not shown). Hence, in TKO retinas, most progenitors that exit mitosis migrate to the GCL and express amacrine rather than RGC markers (the proportion of RGC born at E12.5, E13.5 and E16.5 is shown in Fig. S5).

### Increased apoptosis and abnormal cell division in *Dlx1*/*Dlx2*/*Brn3b*-null retinas

**Fig. 5.**
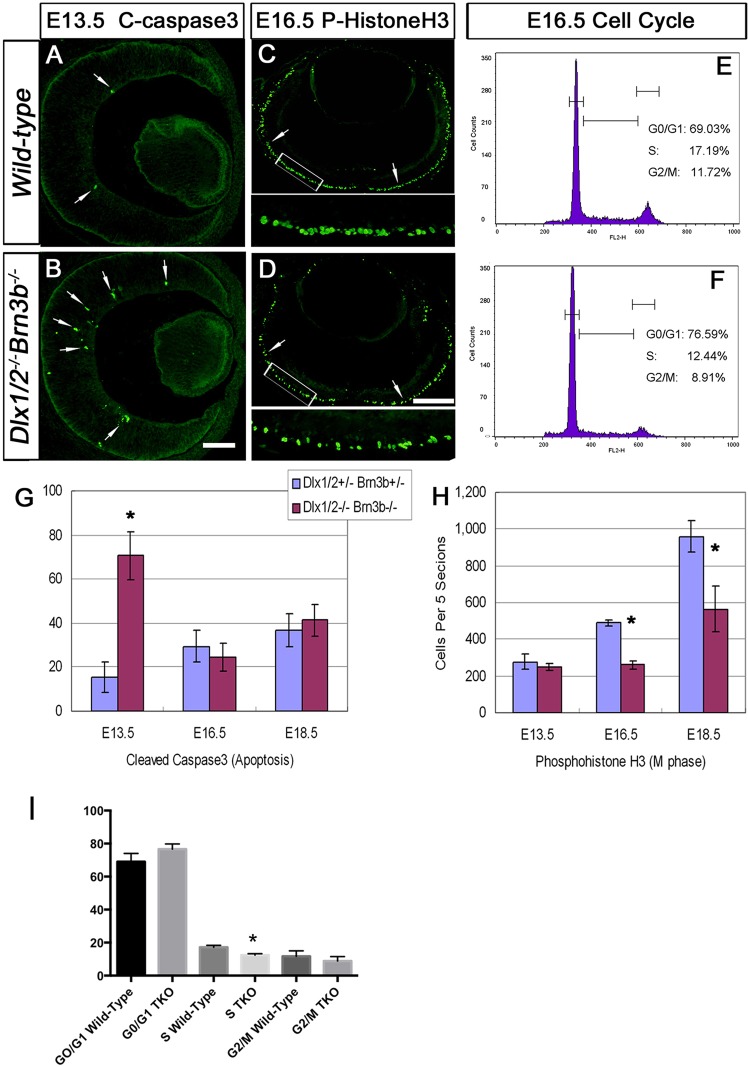
**Combined loss of *Dlx1*/*Dlx2* and *Brn3b* results in enhanced apoptosis and abnormal cell proliferation.** (A,B,G) Cleaved caspase 3 immunostaining shows a fourfold increase in the number of apoptotic cells (arrows) in E13.5 TKO retinas (B,G). There is no significant difference in apoptosis at E16.5 and E18.5 (G). (C,D,H) Anti-phospho-histone H3 quantification revealed a decreased number of cells in M-phase at E16.5 and E18.5 in the TKO (D,H) but not at E13.5 (H). The boxed regions in C and D are shown at higher magnification beneath. (E,F,I) There are lower percentages of S-phase (*P*=0.005) and G2/M-phase (NS) cells at E16.5 in the TKO (*n*=3). Histograms represent the mean±s.d., *n*=4, *P<0.01. Scale bars: 50 μm in B; 25 μm in D.

At E13.5, the TKO had a significant fourfold increase in apoptotic cells (70.5±11 of mutants versus 15.5±7 of wild type, *P*<0.01, *n*=4; Fig. 5A,B,G). However, for E16.5 and E18.5 TKO, apoptotic cell numbers were similar to wild type (Fig. 5G). In the TKO at E13.5, the majority of caspase-3+ cells were confined to the inner retina, where prospective RGCs are located. Unlike the *Brn3b* SKO or *Dlx1*/*Dlx2* DKO, enhanced apoptosis later than E13.5 was not detected in the TKO.

TKO retinas displayed a significant reduction (46% and 41%) in the number of M-phase cells at E16.5 (Fig. 5D,H) and E18.5, respectively (Fig. 5H). Similarly, fewer S-phase cells were detected in E16.5 and E18.5 mutants (data not shown). Flow cytometry yielded concordant results at E16.5 and E18.5, with significantly reduced proportions of cells in S phase (*P*=0.005, *n*=3) but changes in G2/M and G1/G0 phases were not significant (Fig. 5E,F,I; data not shown).

### Expression of BRN3B and DLX2 in *Atoh7*-null retinas

**Fig. 6.**
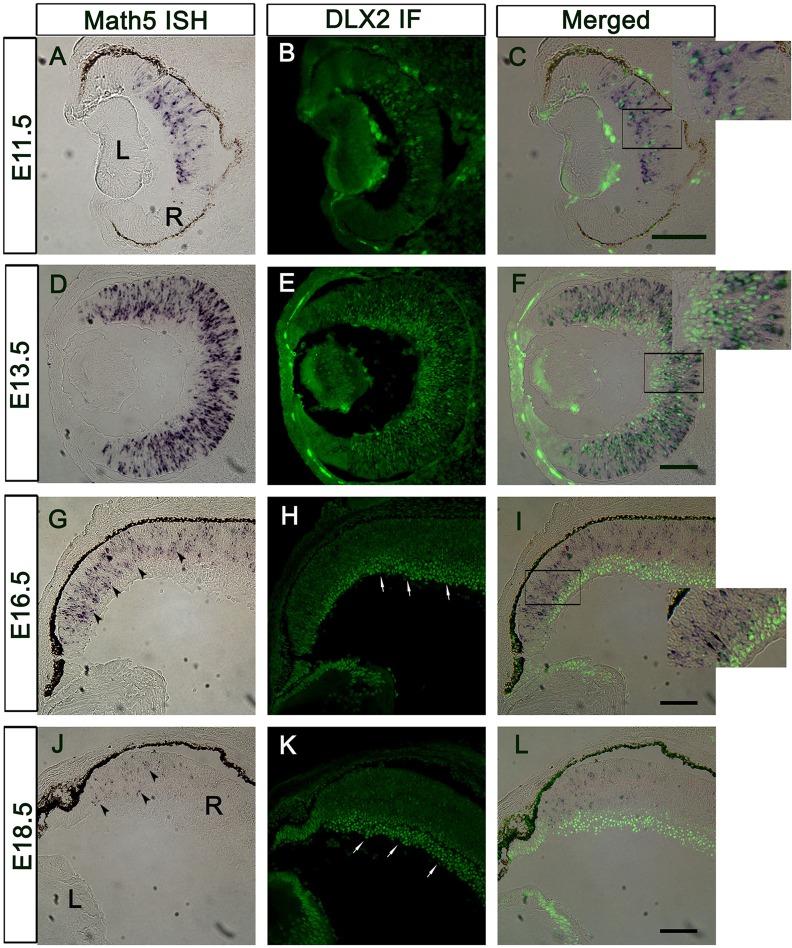
***Atoh7*** **mRNA and DLX2 protein are co-expressed at early but not late embryonic stages.** (A-C) *Atoh7* mRNA and DLX2 protein are expressed in the central E11.5 retina. (D-F) At E13.5, *Atoh7* mRNA is expressed throughout the retina, except the inner central region; *Atoh7*+/DLX2+ cells are located in the middle layer. *Atoh7*−/DLX2+ cells are easily detected in the inner central retina, where differentiated cells are located. *Atoh7*+/DLX2− cells are restricted to the outmost proliferating zone. (G-I) *Atoh7* expression is downregulated at E16.5 (G, arrowheads). DLX2 is strongly expressed in the GCL and inner NBL (H, arrows). Double-positive cells can be detected in the NBL (I, inset, arrows). (J-L) At E18.5, *Atoh7* is localized proximal to the ciliary margin (J, arrowheads). DLX2 expression is restricted in the GCL and inner NBL (K, arrows), without double-positive cells. Scale bars: 100 µm.

**Fig. 7.**
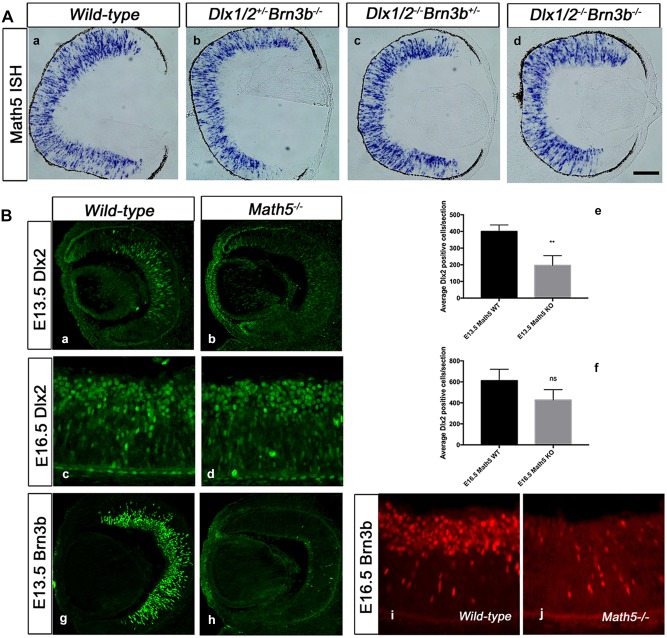
***Atoh*7** **expression is not affected by the combined deletion of *Dlx1*/*Dlx2* and *Brn3b*.** (A) *In situ* hybridization at E13.5 shows *Atoh7* expression throughout the proliferating zone of developing retina, except for the ciliary margin. No difference was detected between wild type and the genetic mutants. Scale bar: 40 μm. (B) DLX2 and BRN3B expression is downregulated in *Atoh7*-null retinas at E13.5. (a-f) DLX2 expression is significantly reduced in the *Atoh7* KO at E13.5 (a,b,e) but not at E16.5 (c,d,f). (G-J) BRN3B levels are severely reduced in the *Atoh7*^−/−^ retina at both E13.5 (g,h) and E16.5 (i,j).

*Atoh7*/*Dlx2* co-expressing cells are present at E11.5 when DLX2 is first detected (Fig. 6A-C), extending to E13.5 (Fig. 6D-F), but co-expression is absent at E18.5 (Fig. 6G-I, E16.5; J-K, E18.5). *Atoh7* expression was unaffected in the *Brn3b* SKO, *Dlx1/Dlx2* DKO and the TKO (Fig. 7A). BRN3B expression was severely reduced in the *Atoh7*^−/−^ retina at E13.5 (Fig. 7Bg,h) and E16.5 (Fig. 7Bi,j). However, DLX2 expression was significantly reduced at E13.5 (*P*=0.01, Fig. 7Ba,b,e) but not significantly in the *Atoh7*-null GCL at E16.5, a time when *Atoh7* expression is downregulated (*P*=0.09, Fig. 7Bc,d,f). Interestingly, of the few BRN3B+ cells remaining in the *Atoh7*^−/−^ GCL at E16.5, a proportion co-express DLX2 (data not shown), supporting a role for DLX2 in promoting or maintaining *Brn3b* expression in the absence of *Atoh7* function.

### DLX1/DLX2 regulate *Brn3b* by specific binding to the *Brn3b* promoter *in vivo*

**Fig. 8.**
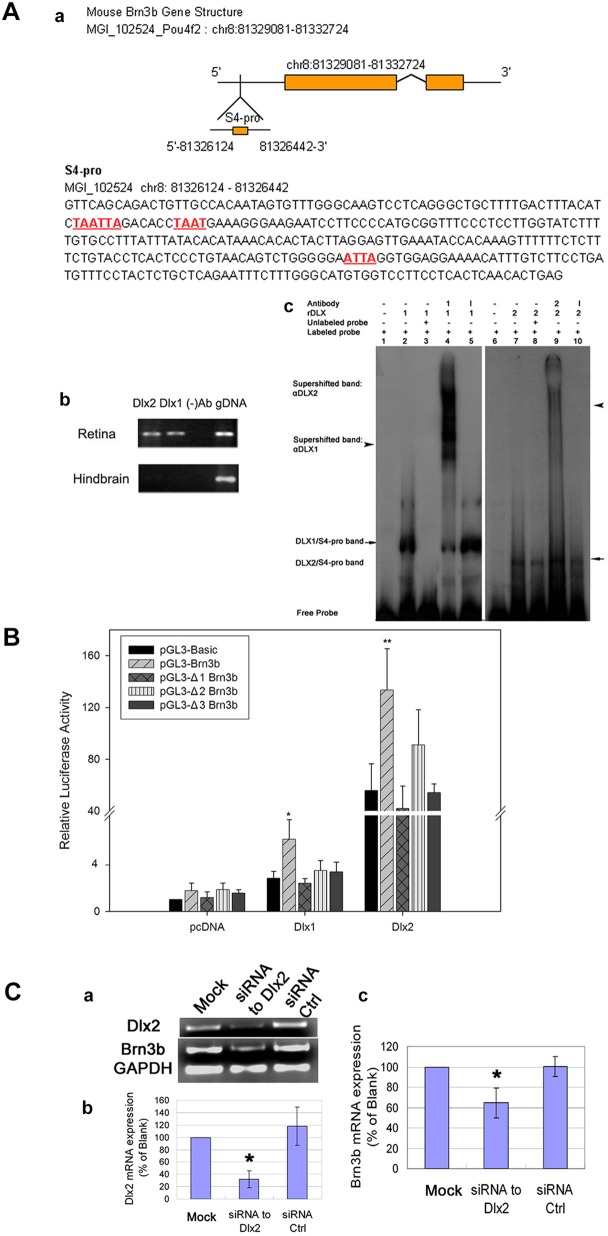
**DLX1 and DLX2 regulate *Brn3b* expression.** (A) DLX1 and DLX2 proteins bind to the *Brn3b* promoter region. (a) *Brn3b* has candidate homeodomain binding sites located within the 5′-promoter region (denoted 3bP4) (a, underlined). (b) ChIP identified specific *Brn3b* promoter DNA sequences occupied by DLX1/DLX2 *in vivo* (lanes 1, 2). E16.5 hindbrain was a negative tissue control. Control ChIP was performed without primary antibodies (lane 3). Genomic DNA was used as positive input (lane 4). (c) Using recombinant (r) DLX1 and DLX2 proteins, EMSA demonstrated direct and specific *in vitro* binding of DLX1/DLX2 proteins to the *Brn3b* promoter region (3bP4) identified by ChIP *in situ*. Radiolabeled 3bP4 oligonucleotides, when incubated alone, migrate as free probe (lanes 1, 6). rDLX1 or rDLX2 proteins form complexes with labeled 3bP4 probe and are shifted (lanes 2, 7 arrows, respectively). Specific DLX1/DLX2 antibodies, when incubated with rDLX1/rDLX2 proteins and labeled 3bP4 probe, form supershifted bands (lanes 4, 9 arrowheads, respectively). Excess unlabeled probe was used for cold competition (lanes 3, 8). An antibody to mouse IgG was used as a control without supershifts (lanes 5, 10). (B) DLX1/DLX2 activate *Brn3b* transcription *in vitro*. Co-transfection of either *Dlx1* or *Dlx2* with the *Brn3b* promoter region 3bP4 reporter construct activated luciferase activity. Mutations of any of the three candidate binding motifs significantly reduced the activation of reporter gene activity by DLX1 and DLX2. *P<0.05, **P<0.01. (C) Knockdown of *Dlx2* expression in primary embryonic retinal cultures at E14.5 results in decreased *Brn3b* expression. (a) RT-PCR shows reduced expression of *Dlx2* and *Brn3b* mRNA in the *Dlx2* siRNA transfected cells, compared with untreated and control siRNA transfected cells. (b,c) Quantitative real-time RT-PCR demonstrated efficient knockdown of *Dlx2* expression using specific siRNA but not with control siRNA. *Dlx2* knockdown results in a significant reduction in *Brn3b* expression (*P<0.05).

As development proceeds, more BRN3B-positive cells express DLX2 and, from E16.5, all BRN3B+ cells co-express DLX2 (Fig. S1). We postulated that DLX1 and/or DLX2 regulate *Brn3b* transcription during retinogenesis. We used chromatin immunoprecipitation (ChIP) of embryonic retina and specific antibodies to detect DLX proteins localized to the *Brn3b* promoter region *in vivo.* Candidate regions were selected based on groups of putative TAAT/ATTA homeodomain consensus DNA-binding motifs. We focused on a region of the *Brn3b* promoter 2263 bp upstream of the start codon. ChIP was performed by using PFA crosslinked cells prepared from E16.5 retina with hindbrain used as a negative tissue control (Zhou et al., 2004). For PCR analysis, we designated seven fragments as 3bP1-3bP7. Only site 3bP4 was amplified from DLX1- or DLX2-immunoprecipitated DNA, confirming DLX1 and DLX2 occupancy of this *Brn3b* promoter region in E16.5 retina (Fig. 8Aa,b).

Radiolabeled 3bP4 oligonucleotide fragments were incubated with recombinant DLX1/DLX2 proteins; we observed two specific protein-DNA band shifts as DLX1-3bP4 and DLX2-3bP4 complexes in electrophoretic mobility shift assays (EMSA) (Fig. 8Ac, lanes 2,7). These bands were competitively inhibited by unlabeled 3bP4 probe (Fig. 8Ac, lanes 3,8), and were ‘supershifted’ by addition of specific DLX1 or DLX2 antibodies (Fig. 8Ac, lanes 4,9). IgG was used as a control antibody (Fig. 8Ac, lanes 5,10).

We then performed transient co-transfection and site-directed mutagenesis assays. A plasmid expressing *Dlx1* or *Dlx2*, or both, was co-transfected into HEK293 cells with a vector in which the *Brn3b* 3bP4 promoter region drives reporter gene expression. DLX1 and DLX2 co-transfection resulted in 2.1- and 2.5-fold increases of luciferase activity, respectively (Fig. 8b). Co-transfection of *Dlx1* with *Dlx2* yielded similar results to *Dlx2* co-transfection alone (Fig. S4). Mutations of any of the three candidate binding motifs within the 3bP4 promoter region significantly reduced luciferase activity by DLX1 and DLX2 *in vitro*, suggesting that DLX1- or DLX2-mediated activation of 3bP4 promoter expression may occur via any one of these binding sites.

### *Brn3b* expression is decreased by *Dlx2* knockdown in primary embryonic retinal cultures

Primary cultures of wild-type E14.5 retina were transiently transfected with siRNA targeting the *Dlx2*-coding sequence or a scrambled control siRNA (de Melo et al., 2008). There was efficient knockdown of *Dlx2* mRNA, with a concomitant decrease of *Brn3b* mRNA expression, compared with the transfection of control siRNA and untreated cells (Fig. 8c). Taken together with the RGC phenotype of the *Dlx1*/*Dlx2* DKO (de Melo et al., 2005), these knockdown experiments support that *Dlx2* function is necessary for *Brn3b* expression *in situ*.

### Gain of *Dlx2* function *in utero* results in ectopic *Brn3b* expression *in vivo*

**Fig. 9.**
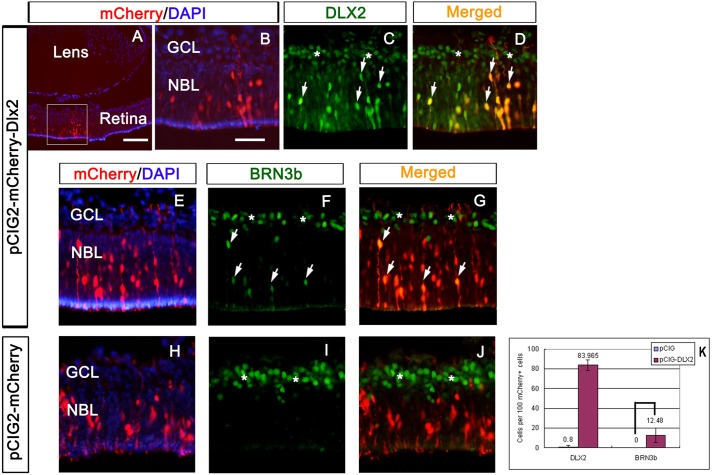
***In utero* electroporation of *Dlx2* results in ectopic *Brn3b* expression *in vivo*.** E14.5 retinas electroporated with pCIG2-mCherry-*Dlx2* (A-G) and pCIG2-mCherry control (H-J) plasmids were harvested at E18. In pCIG2-mCherry-*Dlx2* electroporated retinas, mCherry epifluorescence replicated ectopic DLX2 expression in the outer NBL (arrows in C,D), resulting in ectopic BRN3B expression in the outer NBL (arrows in F,G); results are quantified in K. Endogenous DLX2 and BRN3B expression was also detected in the GCL and INL (stars in C,D,F,G,I,J). No ectopic BRN3B expression was detected in control electroporated NBL (I,J). Scale bars: 50 μm in A; 20 μm in B-J.

Gain-of-function assays in retinal explants are compromised by the loss of trophic support of RGCs due to optic nerve transection during tissue preparation (de Melo et al., 2008). Subsequently, we ectopically expressed *Dlx2* in the intact embryo. E14.5 retinas were electroporated *in utero* with pCIG2-mCherry-*Dlx2* (Fig. 9A-G) or pCIG2-mCherry control plasmids (Fig. 9H-J) then harvested at E18. In pCIG2-mCherry-*Dlx2* electroporated retinas, mCherry epifluorescence reflects ectopic DLX2 expression in the outer NBL (arrows in Fig. 9C,D) with ectopic BRN3B expression in the outer NBL (arrows in Fig. 9F,G). Endogenous DLX2 and BRN3B expression was also detected in the GCL and INL (stars in Fig. 9C,D,F,G,I,J). No ectopic BRN3B was detected in the control NBL (Fig. 9I,J). Similar results were obtained from additional experimental (3) and control (2) electroporated retinas. To distinguish from endogenous DLX2 or BRN3B expression, cell counting was performed on mCherry+ cells located in the outer NBL but not in the GCL. Eighty-four percent of mCherry+ cells express DLX2 in *Dlx2* electroporated retinas. Twelve percent of these mCherry+ cells co-express BRN3B in *Dlx2* but not in control electroporated retinal patches *in vivo*. *P*<0.01, *n*=4 (Fig. 9K). These *in utero* gain-of-function experiments support that exogenous *Dlx2* gene function is sufficient for *Brn3b* expression in the embryonic retina *in vivo*.

### Regulation of *Dlx2* expression by BRN3B is not mediated by direct interactions with *Dlx1*/*Dlx2* regulatory regions *in vivo*

*Brn3b* negatively regulates *Dlx1* and *Dlx2* in the embryonic retina (Mu et al., 2005; Qiu et al., 2008). We observed a transient increase in DLX2 expression at E13.5 but not at E11.5 or E16.5 in the *Brn3b* SKO retina (Fig. S6). As RGC apoptosis occurs in *Brn3b* mutants at ∼E15.5, the initial increase in DLX2 expression could be lost due to the ensuing cell death. Repression of *Dlx2* by BRN3B could occur earlier, during the peak of early born RGC differentiation regulated by *Atoh7-Brn3b*. This repression is removed with diminishing *Atoh7* expression after E16.5 (Brown et al., 1998).

We tested whether this negative regulation could be due to occupancy of *Dlx1*/*Dlx2* regulatory regions by BRN3B. *Cis*-acting elements of the *Dlx1*/*Dlx2* bigenic cluster include two intergenic enhancers, I12b and I12a, and two upstream regulatory elements (URE), URE1 and URE2 (Ghanem et al., 2003, 2007) (Fig. S7). These elements were examined for consensus *Pou*4f2 DNA-binding sites [5′-(A/G)TTAATGAG(C/T)-3′] (Xiang et al., 1995); two putative binding sites are in URE2 with one site in I12b. Two putative *Pou4f2*-binding sites were also found in *Dlx1* exon 3 and *Dlx2* exon 2. BRN3B directly regulates expression of *Eomes*, a T-box TF (Mao et al., 2008a). BRN3B occupancy was not detected at any of these four *Dlx1*/*Dlx2 cis*-regulatory regions, although we replicated binding to the *Eomes* promoter (Fig. S7). Hence, repression of *Dlx1*/*Dlx2* expression by BRN3B is likely mediated by protein-protein interactions (Feng et al., 2011) rather than transcriptional regulation.

## DISCUSSION

### Downstream of ATOH7, *Dlx1*/*Dlx2* and *Brn3b* are necessary for RGC specification

Neither *Dlx1/Dlx2* nor *Brn3b* is necessary for the initiation of RGC differentiation. Expression of TFs positioned upstream of *Dlx1*/*Dlx2* or *Brn3b*, such as *Atoh7* and *Vsx2* (*Chx*10), is required for the competence of RPC to initiate expression of *Brn3b* and *Dlx1*/*Dlx2* for terminal differentiation, maturation and survival of RGCs (Wang et al., 2001; Yang et al., 2003). DLX1/DLX2 expression originates in *Chx10*+ cells (de Melo et al., 2003); similarly, using lineage tracing in the zebrafish retina, *Ath*5-expressing progenitors originate from Vsx2+ RPC (Jusuf et al., 2011; Vitorino et al., 2009).

The observed 70% loss of RGCs in *Brn3b* KO mice occurs at E15.5 or later, whereas 33% loss of RGCs in the *Dlx1*/*Dlx2* DKO mice is detected by E18.5 (de Melo et al., 2005; Gan et al., 1999). Unlike these ‘late’ RGC losses, we found that the TKO resulted in a dramatic reduction of RGCs by E12.5. Although most E12.5 and E13.5 birth-dated retinal cells were in the GCL, few expressed RGC differentiation markers in the TKO. In addition to increased apoptosis in the TKO, decreased proliferation, as demonstrated by decreased staining for phospho-H3 (M-phase marker) and reduced S-phase (by FACS analysis), could also contribute to the observed dramatic RGC loss. Potentially, this cell cycle phenotype in the TKO could be due to increased expression of cell cycle regulators such as p53, CDKN1B (p27) or CDKN1C (p57). Hence, without affecting early migration of RPC to the nascent GCL, combined loss of *Dlx1*/*Dlx2* and *Brn3b* function severely impaired RGC genesis, proliferation, differentiation and survival.

**Fig. 10.**
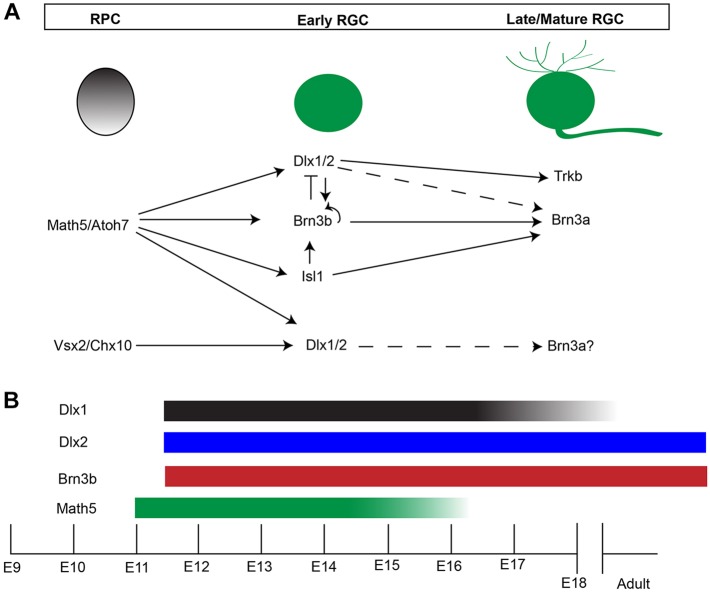
**A model for transcriptional regulation of RGC differentiation.** (A) Loss of DLX2-expressing cells in E13 *Atoh7* KO supports *Dlx1*/*Dlx2* participation in a regulatory pathway downstream of *Atoh7*, where, along with *Brn3b* and *Isl1*, *Dlx1*/*Dlx2* then co-regulates the genes required for RGC differentiation and survival. In addition, cross-regulation of these factors is observed. Loss of *Dlx2*, *Brn3b* or *Isl1* individually does not affect initial RGC genesis, suggesting that a combination/co-regulation of these factors and/or other unidentified factors are responsible for RGC fate specification. In later development, loss of *Dlx2*, *Brn3b* and *Isl1* all result in increased RGC death, supporting the requirement of these factors for RGC survival by controlling the expression of genes necessary for this process, including transcriptional control of *Brn3a* by DLX2 (Q.Z. and D.D.E., unpublished), ISL1 and BRN3B (Pan et al., 2008), and DLX2-mediated regulation of *Trkb* (de Melo et al., 2008). Solid lines, established regulation; broken lines, proposed regulation. (B) *Atoh7* expression begins at ∼E11, preceding *Dlx1*/*Dlx2* and *Brn3b*. *Atoh7* and *Dlx1* expression are both downregulated after E16.5, with neither detected after P0. *Dlx2* and *Brn3b* expression starts at E11.5 and persists to adulthood.

In *Isl1*/*Brn3b* DKO mice, there was severe RGC loss but normal RGC genesis before E13.5, and with *Brn3b*, *Isl1* was shown to define a distinct, yet overlapping, RGC subpopulation under the regulation of ATOH7 (Mu et al., 2008; Pan et al., 2008). Almost complete RGC loss, as well as severe losses of other retinal cell types, resulted in *Atoh7*/*Brn3b* DKO mice (Moshiri et al., 2008). Based upon co-expression of DLX2 with *Atoh7* or BRN3B as early as E11.5, reduced DLX2 expression in the *Atoh7* KO at E13.5 (Fig. 7) and report of a *Dlx* regulatory region as a direct ATH5 target in the chick retina (Del Bene et al., 2007), we propose that *Atoh7-Brn3b/Isl1* and *Atoh7*-*Dlx1*/*Dlx2*-*Brn3b* regulate parallel transcriptional pathways for RGC differentiation (Fig. 10). *Atoh7* and *Dlx2* co-expressing RPCs could later co-express DLX2/BRN3B. Furthermore, downregulation of DLX2 by BRN3B in some RGC progenitors may be a mechanism underlying RGC subtype specification. Lineage tracing and evaluation of cadherin 6 (De la Huerta et al., 2012) and other markers will contribute towards identifying a role for specific RGC subtypes. Deletion of both *Isl1* and *Brn3b* would not affect RPC competence to become RGCs, as *Dlx1*/*Dlx2* would be available for promoting RGC differentiation. However, deletion of *Dlx1*/*Dlx2* and *Brn3b* would block two of the more dominant pathways for RGC differentiation and survival. The few remaining RGC in *Dlx1*/*Dlx2*/*Brn3b* compound mutants could derive from the small population of *Isl1*-expressing progenitors (Fig. 1Bh), which are distinct from those expressing *Brn3b* (Moshiri et al., 2008), although ISL1 also co-labels ChAT+ amacrine cells. Interestingly, these early born cholinergic amacrine cells also derive from *Ath*5-lineage cells (Jusuf et al., 2012). ISL1 and BRN3B can cooperate to specify RGC cell fate (Li et al., 2014; Wu et al., 2015).

In addition to early RGC loss, we found a significant increase in dislocated amacrine cells in the E18.5 TKO GCL. This result was unexpected, as only RGCs are affected by deletion of either *Dlx1*/*Dlx2* or *Brn3b* (de Melo et al., 2005; Erkman et al., 1996; Gan et al., 1996). A similar phenotype was reported in *Atoh7* mutants, with increased cones, bipolar and Müller cells, and increased starburst amacrine cells in the GCL (Brown et al., 2001; Wang et al., 2001). *Atoh7*, which is expressed in RPC, promotes RGC specification by driving cell cycle exit and repressing non-RGC specifying TFs (Brown et al., 1998, 2001; He et al., 2012; Jusuf et al., 2012; Le et al., 2006; Mu et al., 2008). However, lineage tracing of *Atoh7*+ cells in the mouse also suggests a more indirect role for *Atoh7* in RGC specification (Brzezinski et al., 2012).

Amacrine cell specification in TKO retinas might result from a different mechanism than that found in the *Atoh7* mutants. There was preserved expression of *Atoh7* and NEUROD1 in the *Brn3b* KO, *Dlx1*/*Dlx2* KO and TKO mice. RPCs may accumulate more than one competency by expressing ‘early’ factors, such as *Atoh7* or *Neurod1*. Using lineage tracing, *Atoh7* expression is associated with RGC, photoreceptors, horizontal and amacrine cells (Yang et al., 2003). Cells with restricted developmental potentials migrate properly and are further specified to differentiate under the regulation of factors expressed later in retinogenesis. In this case, both *Dlx1*/*Dlx2* and *Brn3b* could act downstream of *Atoh7*, promoting the terminal differentiation of RGCs (Fig. 10). Without *Dlx1*/*Dlx2* and *Brn3b*, cells restricted to RGC and amacrine cell fates migrate to the GCL but are unable to terminally differentiate into RGCs and either undergo apoptosis or differentiate into amacrine cells.

Interestingly, there is an increase in ISL+ cholinergic amacrine cells in a *Atoh7*-*Cre* knock-in mouse (Feng et al., 2010). Similarly, the *Barhl2* KO retina demonstrates a twofold increase in ChAT+ amacrine cells and 35% RGC cell death (Ding et al., 2009). A *Neurod1* knock-in at the *Atoh7* locus re-established RGC gene expression, specification and optic nerve formation, without over-producing amacrine cells (Mao et al., 2008b). RPC single cell gene-expression profiles showed significant levels of *Atoh7*, *Neurog2* and *Neurod1* in individual RPCs (Trimarchi et al., 2008). Hence, ‘early’ factors, including *Atoh7* and *Neurod1*, advance RPC to more narrowly defined but overlapping competencies, whereas, ‘late’ factors, such as *Dlx1*/*Dlx2* and *Brn3b*, promote these restricted-potential RPCs towards more specialized fates.

### *Dlx1*/*Dlx2* and *Brn3b* function in co-regulatory genetic pathways

Spatiotemporal DLX2 and BRN3B expression studies supported a second, later phase of *Brn3b* expression regulated by DLX1/DLX2. Both DLX1 and DLX2 bind to a specific *Brn3b* promoter region *in situ* and activate its expression *in vitro*. Knockdown of *Dlx2* in primary cultures resulted in decreased expression of *Brn3b*, consistent with reduced BRN3B expression in the *Dlx1*/*Dlx2* DKO (de Melo et al., 2005). Ectopic expression of BRN3B occurred following *in utero* retinal electroporation of *Dlx2*. These observations strongly support the direct regulation by DLX1 and DLX2 of *Brn3b* transcription *in vivo*. Hence, *Dlx1* and/or *Dlx2* are necessary and sufficient for *Brn3b* expression during retinal development.

Loss of *Dlx1*/*Dlx2* function results in reduced expression of *Pou3f1* and *Pou3f4* in the embryonic forebrain (Anderson et al., 1997b), suggesting that DLX1/DLX2 could regulate other Class III POU HD TFs besides *Brn3b*, such as *Brn3a* or *Brn3c* (*Pou4f3*) in the developing retina and might further explain the severe retinal phenotype observed in the TKO. Loss of *Dlx1* and/or *Dlx2* function could reduce transcription of all *Brn3* genes in the retina, therefore blocking the functional redundancy of *Brn3* family members (Pan et al., 2005).

Taken together, the mutual regulatory interactions between *Dlx1*/*Dlx2* and *Brn3b*, and the more severe phenotype observed in the TKO suggests that *Atoh7*-*Dlx1*/*Dlx2*-*Brn3b* and *Atoh7*-*Brn3b*/*Isl1* are two parallel but cross-regulatory genetic pathways in retinogenesis (Fig. 10). Characterization of the *Dlx1* and *Dlx2* transcriptomes will further contribute to understanding their role in retinal development.

## MATERIALS AND METHODS

### Animal and tissue preparation

Animal studies were carried out in accordance with guidelines established by the Canadian Council on Animal Care. *Dlx1/Dlx2* DKO mice were generated as previously described (Anderson et al., 1997a; Qiu et al., 1997). *Brn3b* SKO mice (provided by W.H.K.) were maintained on a CD1 background. The *Dlx1/Dlx2*^+/−^ *Brn3b^+/−^* compound heterozygous line from crossing *Dlx1/Dlx2*^+/−^ heterozygotes with *Brn3b^+/−^* heterozygotes was used to generate *Dlx1/Dlx2/Brn3b* triple homozygous-null (TKO) mice. For comparative studies, all TKO mice were paired with *Dlx1/Dlx2*^+/−^*Brn3b^−/−^* (SKO), *Dlx1/Dlx2*^−/−^*Brn3b^+/−^* (DKO) and *Dlx1/Dlx2*^+/−^*Brn3b^+/−^* (all genetic heterozygotes are wild type) littermate controls. Genotyping was performed as previously described (Gan et al., 1996; Qiu et al., 1997). Embryonic age was determined by the day of appearance of the vaginal plug (E0.5). E16.5 and E18.5 eyes were dissected from embryos whereas E13.5 eyes were left *in situ* prior to brief fixation in 4% paraformaldehyde (PFA) in phosphate-buffered saline (PBS). Tissues were sectioned coronally at 12 μm.

### Histology, immunofluorescence, TUNEL assay and *in situ* hybridization

Histological and immunofluorescence staining on cryosections was performed as described previously (de Melo et al., 2005). Primary antibodies used were: mouse anti-BrdU (1:200, Chemicon), mouse anti-BRN3A (1:200, Santa Cruz), goat anti-BRN3B (1:200, Santa Cruz), rabbit anti-caspase 3 (1:500, Cell Signaling Technologies), rabbit anti-DLX2 (1:400, C199 affinity purified), mouse anti-ISLET1 (1:600, DSHB, University of Iowa), rabbit anti-phosphohistone H3 (1:1000, Upstate), rabbit anti-PROX1 (1:500, Chemicon), rabbit anti-PAX6 (1:800, Covance) and mouse anti-syntaxin (1:6000, Sigma). Secondary antibodies and fluorescent tertiary molecules used were: FITC-conjugated goat anti-rabbit (1:200), biotin-SP-conjugated goat anti-rabbit (1:200), biotin-SP-conjugated goat anti-mouse (1:200) (Jackson ImmunoResearch), streptavidin-conjugated Oregon Green 488 (1:200) and streptavidin-conjugated Texas Red (1:200) (Molecular Probes). Negative controls omitted the primary antibody. TUNEL staining used the In Situ Cell Death Detection Kit, TMR Red (Roche Diagnostics). Non-radioactive digoxigenin *in situ* RNA hybridization was performed as described previously (de Melo et al., 2005).

### Retinal explant and amacrine subtype analysis

Retinas were dissected from E18.5 TKO and wild-type littermates, and transferred onto Millicell-CM cell culture insert with 0.4 μm filters (Millipore). After removing the lens and the hyaloid vessels, retinas were flattened and cultured at 37°C with 5% CO_2_ in a humidified incubator. Explant culture media contained 50% high-glucose MEM, 25% Hank's solution, 25% horse serum, 200 μM L-glutamine, 6.75 mg/ml glucose, 2.5 mM HEPES buffer solution and 1% penicillin/streptomycin. After 7 DIV, explants were fixed in 4% PFA for 30 min and sectioned. ChAT+ cells were counted at 120 μm intervals and results were pooled.

### Pulse labeling and birth-dating

For pulse labeling, BrdU (100 μg/g) was injected intraperitoneally (i.p.) into pregnant dams 1 h before euthanasia. Sections were incubated in 50% formamide/2× SSC for 2 h at 65°C and 2 N HCl for 30 min at 37°C, followed by 0.1 M sodium borate for 10 min at room temperature.

### Propidium iodide (PI) staining and cell cycle analysis

Retinas were dissociated into single cell suspensions in 1× PBS and fixed in 70% ethanol overnight at 4°C. Cells were resuspended in 250 μl propidium iodide (50 μg/ml) and 1 μl RNase (20 μg/ml), and incubated for 15 min at room temperature. Flow cytometry was performed on a FACSCalibur apparatus (Becton Dickinson), and analyzed using BD CellQuest Pro Version 3.5 software.

### Cell counting and statistical analysis

Cell counts were performed on paired wild-type and mutant retinas (de Melo et al., 2005). For RGCs, BRN3A+ and ISL1+ cells located in the GCL were counted for E16.5 and E18.5 retinas; only ISL1 was used to count RGCs at E13.5. Only PAX6+ cells in the inner NBL were counted as amacrine cells; Syntaxin+/DAPI+ cells in the GCL were counted as dislocated amacrine cells; and PROX1+ cells located in the outer NBL were counted as horizontal cells. For BrdU birth-dating studies, the BRN3A+/BrdU+ cells represented RGCs born at the time of BrdU injection; syntaxin+/BrdU+ cells in GCL represent dislocated amacrine cells born at the time of the BrdU pulse. Statistical analyses were performed with SPSS software; the paired *t*-test was used; *P*<0.05 was considered significant.

### Microscopy and imaging

Images were acquired using an Olympus BX51 microscope with a SPOT 1.3.0 digital camera (Diagnostic Instruments) or an Olympus DP70 digital camera. Fluorescent images were acquired using an Olympus IX81 inverted microscope with a Fluoview FV500 confocal laser scanning system (Olympus Optical). Images were processed using Adobe Photoshop software (Adobe Systems) for presentation.

### Chromatin immunoprecipitation and electrophoretic mobility shift assays

E16.5 retina tissues were isolated for ChIP assays as described previously (Zhou et al., 2004). Oligonucleotide primers for PCR amplification were designed according to the *Brn3b* gene promoter sequence (MGI 102524). The primer sets were: S4F, 5′-GTTCAGCAGACTGTTGCCAC-3′ and S4R, 5′-TCCTTCCTCACTCAACACTGAG-3′. The target region (S4-pro) is 318 bp and located at position 81,326,124-81,326,442, on mouse chromosome 8, within the 5′ proximal promoter 2.6 kb upstream of the *Brn3b* transcription start site. Genomic DNA from the E16.5 mouse embryo tail was used as a positive control. PCR products were purified for TOPO TA cloning and sequenced. Oligonucleotide primers for ChIP experiments using embryonic retina and BRN3B antibody (Fig. S7) are provided in the supplementary Materials and Methods.

For EMSA, the S4-pro region was excised from the pCR2.1-TOPO vector (Invitrogen) with *Eco*RI, and labeled with α-P^32^-dATP (PerkinElmer) using the Klenow (large) Fragment of DNA PolI (Invitrogen). The binding reaction mixture contained labeled probes (90,000 cpm), binding buffer [20% glycerol, 5 mM MgCl_2_, 2.5 mM EDTA, 2.5 mM DTT, 250 mM NaCl and 50 mM Tris-HCl (pH7.5)], 1 μg poly-dI-dC and purified recombinant DLX1 or DLX2 protein, and was incubated for 30 min at room temperature. A 100-fold excess of unlabeled probes was used for ‘cold competition’ experiments; rabbit polyclonal DLX1 and DLX2 antibodies were used for ‘supershift’ experiments; and a rabbit polyclonal antibody to mouse IgG (Jackson ImmunoResearch) was used as a negative antibody control.

### Cell culture and reporter gene assays

HEK-293 cells were grown in alpha DMEM (Gibco) with 10% FBS at 37°C with 5% CO_2_. Twenty-four hours prior to transfection, cells were seeded at a density of 1×10^7^ per 36 mm^2^ dish. Lipofectamine 2000 reagent (Invitrogen) was used for transient transfection. Reporter plasmids were constructed by inserting the 318 bp S4-pro fragment into the pGL3-basic vector (Promega). Putative DLX DNA-binding sites in S4-pro were deleted, respectively, by site-directed mutagenesis using the Quick Change Kit (Stratagene); mutations (Δ1, Δ2 and Δ3) were verified by DNA sequencing. Effector plasmids expressing *Dlx1* and *Dlx2* genes were constructed by insertion of a PCR amplified 790 bp *Dlx1* cDNA and 1020 bp *Dlx2* cDNA (Dr J. Rubenstein, University of California at San Francisco, CA, USA) into the pcDNA3 vector (Invitrogen). The plasmid pRSV-β-gal (Promega) was co-transfected to assess transfection efficiency.

### Primary embryonic retinal cell culture and siRNA transfection

E14.5 CD-1 mice retinas were dissociated with Accumax (Sigma-Aldrich) and cultured on poly-L-lysine and laminin-coated six-well plates. Cells were cultured in DMEM/F12 medium supplemented with HEPES, 0.1% BSA, 1% FBS, N2 and B27 (Invitrogen) at 37°C with 5% CO_2_ for 24 h before interfering RNA (siRNA) transfection. Two duplex siRNAs were designed to target the mouse *Dlx2*-coding sequence and a control siRNA was synthesized to scramble the *Dlx2*-coding sequence (Invitrogen). siRNAs were prepared and transfected as previously described (de Melo et al., 2008). RNA was isolated 48 h after transfection.

### Real-time quantitative PCR analysis

RNA was extracted from cultured retinal cells with TRIzol (Invitrogen), and treated with DNase (Sigma). RNA (1 μg) was used to synthesize cDNA with SuperScript II reverse transcriptase (Invitrogen). cDNA (1 μl) was used as a template for gene amplification. Real-time quantitative PCR was performed using the iCycler iQ system (Bio-Rad). mRNA copy numbers were calculated using standard curves generated by using plasmids containing the target sequences. *Gapdh* was used to normalize *Dlx2* and *Brn3b* mRNA expression levels. Primers were: *Dlx2*-F, 5′ cca aac tca ggt caa aat ctg 3′; *Dlx2*-R, 5′ tta gaa aat cgt ccc cgc g 3′; *Brn3b*-F, 5′ gtc ggc agc ctc ctc ttc ttc tgt g; *Brn3b*-R, 5′ gct tct cgc ggt ggg att tct cag; *Gapdh*-F, 5′ ctc atg acc aca gtc cat gc 3′; *Gapdh*-R, 5′ cac att ggg ggg tag gaa cac 3′.

### *In utero* electroporation

Timed pregnant C57/BL6 mice were used for *in utero* electroporation experiments. *Dlx2* cDNAs were cloned into pCIG2, an expression vector containing a CMV/β-actin enhancer/promoter and an IRES-mCherry cassette. Endotoxin-free plasmid DNA for electroporation was generated using a column-based purification system (Qiagen). *In utero* electroporation was performed as described previously (Dixit et al., 2011; Langevin et al., 2007; Mattar et al., 2008). Injection of E13.5 or E14.5 retinas took advantage of the pigmented retinal epithelium (RPE) for targeting; embryos developed until E18.5. Electroporated eyes were identified by mCherry epifluorescence.

## References

[DEV142042C1] Anderson S. A., Eisenstat D. D., Shi L. and Rubenstein J. L. (1997a). Interneuron migration from basal forebrain to neocortex: dependence on Dlx genes. *Science* 278, 474-476. 10.1126/science.278.5337.474 9334308

[DEV142042C2] Anderson S. A., Qiu M., Bulfone A., Eisenstat D. D., Meneses J., Pedersen R. and Rubenstein J. L. R. (1997b). Mutations of the homeobox genes Dlx-1 and Dlx-2 disrupt the striatal subventricular zone and differentiation of late born striatal neurons. *Neuron* 19, 27-37. 10.1016/S0896-6273(00)80345-1 9247261

[DEV142042C3] Badea T. C., Cahill H., Ecker J., Hattar S. and Nathans J. (2009). Distinct roles of transcription factors brn3a and brn3b in controlling the development, morphology, and function of retinal ganglion cells. *Neuron* 61, 852-864. 10.1016/j.neuron.2009.01.020 19323995 PMC2679215

[DEV142042C4] Barnstable C. J., Hofstein R. and Akagawa K. (1985). A marker of early amacrine cell development in rat retina. *Brain Res.* 352, 286-290. 10.1016/0165-3806(85)90116-6 3896407

[DEV142042C5] Belecky-Adams T., Tomarev S., Li H. S., Ploder L., McInnes R. R., Sundin O. and Adler R. (1997). Pax-6, Prox 1, and Chx10 homeobox gene expression correlates with phenotypic fate of retinal precursor cells. *Invest. Ophthalmol. Vis. Sci.* 38, 1293-1303. 9191592

[DEV142042C6] Brown N. L., Kanekar S., Vetter M. L., Tucker P. K., Gemza D. L. and Glaser T. (1998). Math5 encodes a murine basic helix-loop-helix transcription factor expressed during early stages of retinal neurogenesis. *Development* 125, 4821-4833. 9806930 10.1242/dev.125.23.4821

[DEV142042C7] Brown N. L., Patel S., Brzezinski J. and Glaser T. (2001). Math5 is required for retinal ganglion cell and optic nerve formation. *Development* 128, 2497-2508. 11493566 10.1242/dev.128.13.2497 PMC1480839

[DEV142042C8] Brzezinski J. A. T., Prasov L. and Glaser T. (2012). Math5 defines the ganglion cell competence state in a subpopulation of retinal progenitor cells exiting the cell cycle. *Dev. Biol.* 365, 395-413. 10.1016/j.ydbio.2012.03.006 22445509 PMC3337348

[DEV142042C9] Cepko C. L., Austin C. P., Yang X., Alexiades M. and Ezzeddine D. (1996). Cell fate determination in the vertebrate retina. *Proc. Natl. Acad. Sci. USA* 93, 589-595. 10.1073/pnas.93.2.589 8570600 PMC40096

[DEV142042C10] Chow R. L., Snow B., Novak J., Looser J., Freund C., Vidgen D., Ploder L. and McInnes R. R. (2001). Vsx1, a rapidly evolving paired-like homeobox gene expressed in cone bipolar cells. *Mech. Dev.* 109, 315-322. 10.1016/S0925-4773(01)00585-8 11731243

[DEV142042C11] De la Huerta I., Kim I.-J., Voinescu P. E. and Sanes J. R. (2012). Direction-selective retinal ganglion cells arise from molecularly specified multipotential progenitors. *Proc. Natl. Acad. Sci. USA* 109, 17663-17668. 10.1073/pnas.1215806109 23045641 PMC3491461

[DEV142042C12] de Melo J., Qiu X., Du G., Cristante L. and Eisenstat D. D. (2003). Dlx1, Dlx2, Pax6, Brn3b, and Chx10 homeobox gene expression defines the retinal ganglion and inner nuclear layers of the developing and adult mouse retina. *J. Comp. Neurol.* 461, 187-204. 10.1002/cne.10674 12724837

[DEV142042C13] de Melo J., Du G., Fonseca M., Gillespie L.-A., Turk W. J., Rubenstein J. L. R. and Eisenstat D. D. (2005). Dlx1 and Dlx2 function is necessary for terminal differentiation and survival of late-born retinal ganglion cells in the developing mouse retina. *Development* 132, 311-322. 10.1242/dev.01560 15604100

[DEV142042C14] de Melo J., Zhou Q.-P., Zhang Q., Zhang S., Fonseca M., Wigle J. T. and Eisenstat D. D. (2008). Dlx2 homeobox gene transcriptional regulation of Trkb neurotrophin receptor expression during mouse retinal development. *Nucleic Acids Res.* 36, 872-884. 10.1093/nar/gkm1099 18086710 PMC2241891

[DEV142042C15] Del Bene F., Ettwiller L., Skowronska-Krawczyk D., Baier H., Matter J.-M., Birney E. and Wittbrodt J. (2007). In vivo validation of a computationally predicted conserved Ath5 target gene set. *PLoS Genet.* 3, e159 10.1371/journal.pgen.0030159 PMC1988851 17892326

[DEV142042C16] Ding Q., Chen H., Xie X., Libby R. T., Tian N. and Gan L. (2009). BARHL2 differentially regulates the development of retinal amacrine and ganglion neurons. *J. Neurosci.* 29, 3992-4003. 10.1523/JNEUROSCI.5237-08.2009 19339595 PMC2756297

[DEV142042C17] Dixit R., Lu F., Cantrup R., Gruenig N., Langevin L. M., Kurrasch D. M. and Schuurmans C. (2011). Efficient gene delivery into multiple CNS territories using in utero electroporation. *J. Vis. Exp.* 52, pii: e2957 10.3791/2957 PMC3197065 21730943

[DEV142042C18] Eisenstat D. D., Liu J. K., Mione M., Zhong W., Yu G., Anderson S. A., Ghattas I., Puelles L. and Rubenstein J. L. R. (1999). DLX-1, DLX-2, and DLX-5 expression define distinct stages of basal forebrain differentiation. *J. Comp. Neurol.* 414, 217-237. 10.1002/(SICI)1096-9861(19991115)414:2<217::AID-CNE6>3.0.CO;2-I 10516593

[DEV142042C19] Erkman L., McEvilly R. J., Luo L., Ryan A. K., Hooshmand F., O'Connell S. M., Keithley E. M., Rapaport D. H., Ryan A. F. and Rosenfeld M. G. (1996). Role of transcription factors a Brn-3.1 and Brn-3.2 in auditory and visual system development. *Nature* 381, 603-606. 10.1038/381603a0 8637595

[DEV142042C20] Erkman L., Yates P. A., McLaughlin T., McEvilly R. J., Whisenhunt T., O'Connell S. M., Krones A. I., Kirby M. A., Rapaport D. H., Bermingham J. R. et al. (2000). A POU domain transcription factor-dependent program regulates axon pathfinding in the vertebrate visual system. *Neuron* 28, 779-792. 10.1016/S0896-6273(00)00153-7 11163266

[DEV142042C21] Feng L., Xie Z.-H., Ding Q., Xie X., Libby R. T. and Gan L. (2010). MATH5 controls the acquisition of multiple retinal cell fates. *Mol. Brain* 3, 36 10.1186/1756-6606-3-36 21087508 PMC2994854

[DEV142042C22] Feng L., Eisenstat D. D., Chiba S., Ishizaki Y., Gan L. and Shibasaki K. (2011). Brn-3b inhibits generation of amacrine cells by binding to and negatively regulating DLX1/2 in developing retina. *Neuroscience* 195, 9-20. 10.1016/j.neuroscience.2011.08.015 21875655

[DEV142042C23] Gan L., Xiang M., Zhou L., Wagner D. S., Klein W. H. and Nathans J. (1996). POU domain factor Brn-3b is required for the development of a large set of retinal ganglion cells. *Proc. Natl. Acad. Sci. USA* 93, 3920-3925. 10.1073/pnas.93.9.3920 8632990 PMC39460

[DEV142042C24] Gan L., Wang S. W., Huang Z. and Klein W. H. (1999). POU domain factor Brn-3b is essential for retinal ganglion cell differentiation and survival but not for initial cell fate specification. *Dev. Biol.* 210, 469-480. 10.1006/dbio.1999.9280 10357904

[DEV142042C25] Ghanem N., Jarinova O., Amores A., Long Q., Hatch G., Park B. K., Rubenstein J. L. R. and Ekker M. (2003). Regulatory roles of conserved intergenic domains in vertebrate Dlx bigene clusters. *Genome Res.* 13, 533-543. 10.1101/gr.716103 12670995 PMC430168

[DEV142042C26] Ghanem N., Yu M., Long J., Hatch G., Rubenstein J. L. R. and Ekker M. (2007). Distinct cis-regulatory elements from the Dlx1/Dlx2 locus mark different progenitor cell populations in the ganglionic eminences and different subtypes of adult cortical interneurons. *J. Neurosci.* 27, 5012-5022. 10.1523/JNEUROSCI.4725-06.2007 17494687 PMC4917363

[DEV142042C27] He J., Zhang G., Almeida A. D., Cayouette M., Simons B. D. and Harris W. A. (2012). How variable clones build an invariant retina. *Neuron* 75, 786-798. 10.1016/j.neuron.2012.06.033 22958820 PMC3485567

[DEV142042C28] Inoue T., Hojo M., Bessho Y., Tano Y., Lee J. E. and Kageyama R. (2002). Math3 and NeuroD regulate amacrine cell fate specification in the retina. *Development* 129, 831-842. 11861467 10.1242/dev.129.4.831

[DEV142042C29] Jusuf P. R., Almeida A. D., Randlett O., Joubin K., Poggi L. and Harris W. A. (2011). Origin and determination of inhibitory cell lineages in the vertebrate retina. *J. Neurosci.* 31, 2549-2562. 10.1523/JNEUROSCI.4713-10.2011 21325522 PMC3083844

[DEV142042C30] Jusuf P. R., Albadri S., Paolini A., Currie P. D., Argenton F., Higashijima S., Harris W. A. and Poggi L. (2012). Biasing amacrine subtypes in the Atoh7 lineage through expression of Barhl2. *J. Neurosci.* 32, 13929-13944. 10.1523/JNEUROSCI.2073-12.2012 23035102 PMC3475408

[DEV142042C31] Langevin L. M., Mattar P., Scardigli R., Roussigné M., Logan C., Blader P. and Schuurmans C. (2007). Validating in utero electroporation for the rapid analysis of gene regulatory elements in the murine telencephalon. *Dev. Dyn.* 236, 1273-1286. 10.1002/dvdy.21126 17377980

[DEV142042C32] Le T. T., Wroblewski E., Patel S., Riesenberg A. N. and Brown N. L. (2006). Math5 is required for both early retinal neuron differentiation and cell cycle progression. *Dev. Biol.* 295, 764-778. 10.1016/j.ydbio.2006.03.055 16690048

[DEV142042C33] Li R., Wu F., Ruonala R., Sapkota D., Hu Z. and Mu X. (2014). Isl1 and Pou4f2 form a complex to regulate target genes in developing retinal ganglion cells. *PLoS ONE* 9, e92105 10.1371/journal.pone.0092105 24643061 PMC3958441

[DEV142042C34] Livesey F. J. and Cepko C. L. (2001). Vertebrate neural cell-fate determination: lessons from the retina. *Nat. Rev. Neurosci.* 2, 109-118. 10.1038/35053522 11252990

[DEV142042C35] MacNeil M. A. and Masland R. H. (1998). Extreme diversity among amacrine cells: implications for function. *Neuron* 20, 971-982. 10.1016/S0896-6273(00)80478-X 9620701

[DEV142042C36] Mao C.-A., Kiyama T., Pan P., Furuta Y., Hadjantonakis A.-K. and Klein W. H. (2008a). Eomesodermin, a target gene of Pou4f2, is required for retinal ganglion cell and optic nerve development in the mouse. *Development* 135, 271-280. 10.1242/dev.009688 18077589 PMC2893890

[DEV142042C37] Mao C.-A., Wang S. W., Pan P. and Klein W. H. (2008b). Rewiring the retinal ganglion cell gene regulatory network: Neurod1 promotes retinal ganglion cell fate in the absence of Math5. *Development* 135, 3379-3388. 10.1242/dev.024612 18787067

[DEV142042C38] Mattar P., Langevin L. M., Markham K., Klenin N., Shivji S., Zinyk D. and Schuurmans C. (2008). Basic helix-loop-helix transcription factors cooperate to specify a cortical projection neuron identity. *Mol. Cell. Biol.* 28, 1456-1469. 10.1128/MCB.01510-07 18160702 PMC2258790

[DEV142042C39] Milam A. H., Dacey D. M. and Dizhoor A. M. (1993). Recoverin immunoreactivity in mammalian cone bipolar cells. *Vis. Neurosci.* 10, 1-12. 10.1017/S0952523800003175 8424920

[DEV142042C40] Moshiri A., Gonzalez E., Tagawa K., Maeda H., Wang M., Frishman L. J. and Wang S. W. (2008). Near complete loss of retinal ganglion cells in the math5/brn3b double knockout elicits severe reductions of other cell types during retinal development. *Dev. Biol.* 316, 214-227. 10.1016/j.ydbio.2008.01.015 18321480 PMC2483850

[DEV142042C41] Mu X., Fu X., Sun H., Beremand P. D., Thomas T. L. and Klein W. H. (2005). A gene network downstream of transcription factor Math5 regulates retinal progenitor cell competence and ganglion cell fate. *Dev. Biol.* 280, 467-481. 10.1016/j.ydbio.2005.01.028 15882586

[DEV142042C42] Mu X., Fu X., Beremand P. D., Thomas T. L. and Klein W. H. (2008). Gene-regulation logic in retinal ganglion cell development: Isl1 defines a critical branch distinct from but overlapping with Pou4f2. *Proc. Natl. Acad. Sci. USA* 105, 6942-6947. 10.1073/pnas.0802627105 18460603 PMC2383966

[DEV142042C43] Ohsawa R. and Kageyama R. (2008). Regulation of retinal cell fate specification by multiple transcription factors. *Brain Res.* 1192, 90-98. 10.1016/j.brainres.2007.04.014 17488643

[DEV142042C44] Pan L., Yang Z., Feng L. and Gan L. (2005). Functional equivalence of Brn3 POU-domain transcription factors in mouse retinal neurogenesis. *Development* 132, 703-712. 10.1242/dev.01646 15647317

[DEV142042C45] Pan L., Deng M., Xie X. and Gan L. (2008). ISL1 and BRN3B co-regulate the differentiation of murine retinal ganglion cells. *Development* 135, 1981-1990. 10.1242/dev.010751 18434421 PMC2758274

[DEV142042C46] Qiu M., Bulfone A., Ghattas I., Meneses J. J., Christensen L., Sharpe P. T., Presley R., Pedersen R. A. and Rubenstein J. L. R. (1997). Role of the Dlx homeobox genes in proximodistal patterning of the branchial arches: mutations of Dlx-1, Dlx-2, and Dlx-1 and -2 alter morphogenesis of proximal skeletal and soft tissue structures derived from the first and second arches. *Dev. Biol.* 185, 165-184. 10.1006/dbio.1997.8556 9187081

[DEV142042C47] Qiu F., Jiang H. and Xiang M. (2008). A comprehensive negative regulatory program controlled by Brn3b to ensure ganglion cell specification from multipotential retinal precursors. *J. Neurosci.* 28, 3392-3403. 10.1523/JNEUROSCI.0043-08.2008 18367606 PMC2459333

[DEV142042C48] Trimarchi J. M., Stadler M. B. and Cepko C. L. (2008). Individual retinal progenitor cells display extensive heterogeneity of gene expression. *PLoS ONE* 3, e1588 10.1371/journal.pone.0001588 18270576 PMC2220035

[DEV142042C49] Viczian A. S., Solessio E. C., Lyou Y. and Zuber M. E. (2009). Generation of functional eyes from pluripotent cells. *PLoS Biol.* 7, e1000174 10.1371/journal.pbio.1000174 19688031 PMC2716519

[DEV142042C50] Vitorino M., Jusuf P. R., Maurus D., Kimura Y., Higashijima S. and Harris W. A. (2009). Vsx2 in the zebrafish retina: restricted lineages through derepression. *Neural Dev.* 4, 14 10.1186/1749-8104-4-14 19344499 PMC2683830

[DEV142042C51] Voinescu P. E., Kay J. N. and Sanes J. R. (2009). Birthdays of retinal amacrine cell subtypes are systematically related to their molecular identity and soma position. *J. Comp. Neurol.* 517, 737-750. 10.1002/cne.22200 19827163 PMC2814066

[DEV142042C52] Wang S. W., Kim B. S., Ding K., Wang H., Sun D., Johnson R. L., Klein W. H. and Gan L. (2001). Requirement for math5 in the development of retinal ganglion cells. *Genes Dev.* 15, 24-29. 10.1101/gad.855301 11156601 PMC312600

[DEV142042C53] Wu F., Kaczynski T. J., Sethuramanujam S., Li R., Jain V., Slaughter M. and Mu X. (2015). Two transcription factors, Pou4f2 and Isl1, are sufficient to specify the retinal ganglion cell fate. *Proc. Natl. Acad. Sci. USA* 112, E1559-E1568. 10.1073/pnas.1421535112 25775587 PMC4386335

[DEV142042C54] Xiang M. (1998). Requirement for Brn-3b in early differentiation of postmitotic retinal ganglion cell precursors. *Dev. Biol.* 197, 155-169. 10.1006/dbio.1998.8868 9630743

[DEV142042C55] Xiang M., Zhou L., Macke J. P., Yoshioka T., Hendry S. H., Eddy R. L., Shows T. B. and Nathans J. (1995). The Brn-3 family of POU-domain factors: primary structure, binding specificity, and expression in subsets of retinal ganglion cells and somatosensory neurons. *J. Neurosci.* 15, 4762-4785. 7623109 10.1523/JNEUROSCI.15-07-04762.1995 PMC6577904

[DEV142042C56] Yang Z., Ding K., Pan L., Deng M. and Gan L. (2003). Math5 determines the competence state of retinal ganglion cell progenitors. *Dev. Biol.* 264, 240-254. 10.1016/j.ydbio.2003.08.005 14623245

[DEV142042C57] Zhou Q.-P., Le T. N., Qiu X., Spencer V., de Melo J., Du G., Plews M., Fonseca M., Sun J. M., Davie J. R. et al. (2004). Identification of a direct Dlx homeodomain target in the developing mouse forebrain and retina by optimization of chromatin immunoprecipitation. *Nucleic Acids Res.* 32, 884-892. 10.1093/nar/gkh233 14769946 PMC373381

